# Standardization of laboratory practices and reporting of biomarker data in clinical nutrition research

**DOI:** 10.1093/ajcn/nqaa036

**Published:** 2020-05-20

**Authors:** Karen M O'Callaghan, Daniel E Roth

**Affiliations:** Centre for Global Child Health and SickKids Research Institute, Hospital for Sick Children, Toronto, Ontario, Canada; Centre for Global Child Health and SickKids Research Institute, Hospital for Sick Children, Toronto, Ontario, Canada; Department of Paediatrics, Hospital for Sick Children and University of Toronto, Toronto, Ontario, Canada; Department of Nutritional Sciences, Faculty of Medicine, University of Toronto, Toronto, Ontario, Canada

Laboratory-derived measures of nutritional status and related biochemical phenomena (e.g., inflammation and oxidative stress) are critical tools in the nutritional sciences but have well-known challenges and pitfalls (1, 2). Researchers routinely examine biological sources of between- and within-person variation in the analysis of biomarker concentrations (e.g., age, sex, pregnancy, inflammation, etc.). However, close attention to the standardization and validation of laboratory practices is required to reduce the error variation that arises from inconsistencies in specimen handling, assay selection, assay performance, and management and statistical analysis of biomarker data (3–5). Furthermore, complete disclosure of laboratory assay protocols, performance characteristics, and technical limitations is essential to ensure the interpretability of published findings and promote opportunities for coherent pooling of biomarker data in meta-analyses.

In this issue of the Journal, 2 contributions from the Biomarkers Reflecting Inflammation and Nutritional Determinants of Anemia (BRINDA) consortium (6,7) revisit the real-world challenges of inconsistent nutritional biomarker measurement and reporting methods (2). In both studies, investigators used data from multiple population-representative surveys to determine the extent to which biomarkers of micronutrient status (folate and vitamin B12 in 1 study, zinc in the other) are associated with 2 biomarkers of systemic inflammation—C-reactive protein (CRP) and α-1 acid glycoprotein (AGP). As in prior BRINDA studies, the fundamental idea is that if micronutrient and inflammatory markers are consistently correlated, then estimates of the population prevalence of deficiency of that particular micronutrient should include a correction for inflammation (8). However, in both studies, the authors considered the wide variability in laboratory methods used for the measurement of micronutrient biomarkers to be a barrier to pooling of data across surveys (6, 7). For example, Young et al. (6) attributed their decision not to conduct pooled analyses to unquantifiable differences in methods used to assess folate and vitamin B12 status. Similarly, in their application of BRINDA methods to correct zinc concentrations for systemic inflammation, McDonald et al. (7) raised concerns about variability in blood collection procedures and laboratory analyses of plasma zinc, CRP, and AGP concentrations. The decision to forego meta-analyses was reasonable, but the unfortunate consequence was a rather complicated multiplicity of survey-specific analyses. Therefore, while the BRINDA project has undoubtedly made important contributions to our understanding of the role of inflammation in the interpretation of micronutrient biomarker data, it also reminds us of other pervasive and potent sources of variability in micronutrient concentrations—sample collection and storage methods, assay selection and performance, and other laboratory procedures.

The BRINDA authors acknowledged the scant information available to them concerning the specific assays used for each survey included in their studies (6,7). Yet, for several of their surveys, samples were analyzed at the VitMin lab (Juergen Erhardt; http://www.nutrisurvey.de/blood_samples/), which uses a sandwich ELISA method to measure ferritin, retinol-binding protein, soluble transferrin receptor, CRP, and AGP (9). The VitMin lab has been a valued resource in the global micronutrient research community for many years; an initial validation study of its ELISAs was promising (9), although recent comparisons of the VitMin method to a new commercial assay showed poor concordance (10). For the surveys for which samples were analyzed at the VitMin lab, detailed measures of assay technique, measures of precision, and limits of quantification could have been feasibly obtained and assessed as part of the BRINDA project. For example, for CRP—a biomarker of the acute-phase response that is central to many BRINDA analyses—the VitMin lab reports values down to and including zero. According to published reports, prior BRINDA analyses have not routinely taken into account varying precision of the assay at lower concentrations or the VitMin laboratory's stated limit of detection (LOD) of 0.5 mg/L (11–15). The LOD was, however, considered in a limited set of post hoc sensitivity analyses in the 2 recent BRINDA studies in this supplemental issue of the Journal, and was not found to affect their conclusions (6, 7). The LOD—defined as the lowest concentration of an analyte that can be feasibly and consistently detected—refers to the concentration that is reliably distinguished from “analytical noise”; even highly sensitive assays will rarely have the ability to measure concentrations of a true null value (16,17). The lower limit of quantification (LLOQ) may be higher than the LOD and is the lowest concentration that is acceptably quantified by a particular assay, taking into consideration a desired level of accuracy and precision, which typically vary across the assay's reportable range (16,17). For analytes such as CRP, LODs and LLOQs are critically important in epidemiological studies, as considerable proportions of healthy populations can have unquantifiable results even when relatively high-sensitivity assays are used (18). The BRINDA investigators (6,7) were likely faced with a wide range of LODs/LLOQs for CRP assays included in their studies, but for most surveys the LOD/LLOQ was unknown or could only be inferred empirically based on the lowest nonzero value in the dataset (assuming that in generating the dataset, the LLOQ was imputed for all unquantifiable samples). Yet, the implications of variable LLOQs may not be negligible; for example, in a survey from Ecuador, the lowest CRP value in the dataset was 1.9 mg/L, and a majority of preschool children had this value (suggesting that the value was imputed for any child with a CRP value at or below 1.9 mg/L) (7).

As with nearly all laboratory biomarkers, substantial between-assay variations in CRP measurements have prompted unheeded calls for assay standardization (19). To consider how nutritional researchers generally handle the analysis and reporting of CRP, we searched online publications in the *American Journal of Clinical Nutrition* from the latter 6 mo (June to December) of 2019 for articles that reported CRP. Not surprisingly, we found wide variability in CRP assay selection (i.e., manufacturers and platforms/kits) across the 20 studies identified (20–39). All of the named methods were antibody based assays, and most studies used commercially available kits; we found very few (>2) articles that clearly used the same assay, but details about the methods were usually sparse, and 5 of 20 articles (35–39) did not specify the laboratory instrument or assay used. The widespread reliance on antibody-based assays (i.e., immunoassay, ELISA) is common in nutritional research, yet many (if not most) commercial immunoassay/ELISA kits on the market lack adequate validation or standardization (40,41). Reporting of laboratory characteristics, including detection and/or quantification limits and quality control measures, also varied widely among the 20 *American Journal of Clinical Nutrition* articles that reported CRP. Notably, fewer than half (7/20) of the identified articles reported precision estimates or cited prior publications that provided intra- and/or interassay CVs (**Table 1**). Multiple precision estimates across the full range of the data analyzed were rarely described (24,28,33). Some recent articles provide templates for good reporting practice that could be followed by other investigators, such as the succinct but detailed summary of assay performance characteristics presented by Gustafsson et al. (42) and more recently by Hang et al. (43). In these articles, we found that summary tables in the supplementary material enabled relatively complete and transparent reporting of relevant characteristics of the assays and laboratory practices and were particularly useful where several biomarkers were studied.

**TABLE 1:** Reporting of laboratory characteristics for C-reactive protein in original research publications in the *American Journal for Clinical Nutrition* from June to December, 2019^1^

Laboratory assay characteristic	Publications reporting characteristic, *n* (%) (*n *= 20)
LOD and/or LLOQ	4 (20%)
Data handling method below LOD/LLOQ	0
ULOQ	0
Data handling method above ULOQ	0
Inter-assay and/or intra-assay CV	7 (35%)
Specific analyzer and/or assay manufacturer	16 (80%)
Duplicate measurements performed for each sample	2 (10%)

1 LLOQ, lower limit of quantification; LOD, limit of detection; ULOQ, upper limit of quantification.

Very few of the articles reporting CRP that we reviewed provided information about assay limits of sensitivity or the handling of values below such limits (Table 1). Given the uncertainty surrounding values between the LOD and LLOQ (16), the LLOQ is often of more concern in clinical and epidemiological studies because all samples with results below the LLOQ require careful consideration in data analysis. Recognized approaches to handling these samples include the simple substitution of unquantifiable/undetectable results with an arbitrary value (e.g., half the LLOQ) and more sophisticated approaches such as multiple imputation (4). Inappropriate handling of unquantifiables/undetectables (e.g., excluding these samples from the analysis) has the potential to generate biased interpretations of study findings, particularly when there is a high proportion of data below the LLOQ, as may occur with biomarkers that circulate at low systemic concentrations relative to the LLOQ of commonly used assays (4). A recent illustration of thorough reporting of limits of sensitivity can be found in Jones et al. (44), who provided detailed descriptions of LLOQs, substitution of unquantifiable values, and sensitivity analyses. Although LLOQs are more commonly encountered than the corresponding upper limit of quantification (ULOQ), monitoring of nutrient excess may be dependent on an assay's ULOQ. Samples can be readily diluted to measure high concentrations (16,45); however, assay precision may be compromised with serial dilutions, particularly when performed using a solvent other than the original biological matrix (e.g., water rather than serum).

The extent to which variations (or outright errors) in laboratory practices and assays affect inferences in nutritional research seems relatively unknown and probably underappreciated, which is particularly concerning in an era in which public confidence in nutritional research is fragile (46). In addition to efforts to formally standardize assay selection and laboratory practices (47,48), open communication between laboratory personnel and the investigators who analyze the data is essential to ensure that data management and analysis appropriately accounts for assay characteristics, including LODs and LOQs. Peer-reviewed journals could encourage improved practices by instituting checklists and guidelines for describing specimen handling and laboratory assays, or even consider minimum reporting requirements of laboratory-related parameters and performance (**Table 2**). Yet, reporting of standards can only go so far, and greater attention to the optimization and standardization of laboratory activities is essential to promote the validity and reproducibility of clinical and epidemiological research.

**TABLE 2:** Assay quality and performance indicators that may be considered standard reporting requirements of laboratory practices and characteristics in nutritional research^1^

Category	Definition	Explanations and examples
Protocols for specimen collection and handling and laboratory procedures	Detailed outline of procedures and materials sufficient to enable another investigator to replicate the analysis.	Specimen information should include special considerations where appropriate (e.g., trace mineral–free blood collection materials) and details of specimen storage relevant to analyte stability (e.g., number of freeze–thaw cycles). Specific information about commercial kits should include the manufacturer and product number. Detailed protocols and procedures, including QA and QC methods, may be included in supplemental file(s).
LOQs and reportable range	LLOQ and ULOQ-lowest and highest concentrations, respectively-of analyte that can be repeatedly measured with acceptable accuracy and precision (17). Reportable range is the range of values across which results may be quantified and reported for a specific assay in a particular laboratory, including values generated by any standardized pretreatment procedures (e.g., sample dilution) (16).	LLOQ typically refers to the concentration of lowest standard on the calibration curve. LLOQ is distinguished from the LOD, which is lowest concentration of analyte that can be reliably and feasibly differentiated from an acknowledged blank concentration. LLOQ can be ≥LOD but not <LOD (17). Approaches for defining, imputing, or otherwise handling values above/below LOD/LLOQ and ULOQ should be reported.
Precision	Closeness of individual repeated measurements of the same sample, usually described empirically as a measure of imprecision (45), and determined by both within- and between-assay comparisons of results of 2 or more replicates.	SDs and CVs (inter- and intra-assay) of individual repeated measurements under controlled conditions may be used to express precision. CVs may be used to convey within-run as well as between-run variation across batches, personnel, etc. Single CV values for each analyte are less informative than multiple estimates spanning detectable or clinically relevant ranges (e.g., low-, medium- and high-concentration control materials).
Accuracy	Extent to which assay produces “true” results relative to the gold-standard. Bias is average systematic difference between the test result obtained and accepted reference value; also known as systematic measurement error, as distinguished from random error (49).	Accuracy/bias is typically estimated by use of external reference material for which a “true” assigned value is known for the sample. Generally accepted range for variation from true value is ≤5%.
Participation and performance in external quality assessment program	Where applicable, participation in accuracy-based performance testing and/or external quality assurance schemes is encouraged and should be reported.	Results of any proficiency tests should be reported, e.g., VITAL-EQA program (48), DEQAS (50).

1 DEQAS, Vitamin D External Quality Assessment Scheme; LLOQ, lower limit of quantification; LOD, limit of detection; LOQ, limit of quantification; QA, quality assurance; QC, quality control; ULOQ, upper limit of quantification; VITAL-EQA, Vitamin A Laboratory—External Quality Assurance.
